# Postnatal genetic umbilical cord analysis for earliest possible detection of inherited hearing impairment

**DOI:** 10.1007/s00405-023-07986-y

**Published:** 2023-04-24

**Authors:** Manuel Christoph Ketterer, Ralf Birkenhäger, Rainer Beck, Susan Arndt, Antje Aschendorff, Mirjam Kunze

**Affiliations:** 1 Department of Otorhinolaryngology–Head and Neck Surgery, Faculty of Medicine, Medical Center–University of Freiburg, University of Freiburg, Killianstrasse 5, 79106 Freiburg, Germany; 2 Molecular Biological Laboratory, Section for Clinical and Experimental Otology, University Medical Center Freiburg, Freiburg, Germany; 3 Department of Obstetrics and Gynecology, Medical Center, University of Freiburg, Freiburg, Germany

**Keywords:** Cochlear implant, Genetical analysis, Hearing impairment

## Abstract

**Introduction:**

The most common sensorineural disorder in humans is hearing impairment and approximately 60% of prelingual hearing disorders are genetic. Especially parents with a congenital deaf child want to know as early as possible whether their second born child has the same genetic defect or not. The aim of this study is to demonstrate that postnatal genetic umbilical cord analysis is both the earliest detection possibility and sufficient.

**Methods:**

We included first born children with severe hearing impairment that underwent cochlear implantation. All included patients were analyzed genetically and exhibited mutations of either DFNB1 loci or SLC26A4 gene. Additionally, the umbilical cord of the sibling underwent genetic analysis to detect hereditary genetic mutations as early as possible.

**Results:**

49 newborn children out of 22 families were included in this study. Genetic analysis revealed clinical relevant mutations in all first born children and in four siblings via umbilical cord analysis. All patients who have been diagnosed with a relevant genetic mutation that caused severe hearing impairment underwent hearing rehabilitation via cochlear implant surgery.

**Conclusion:**

This study demonstrates the sufficient and early as possible detection of known genetically hearing disorders via umbilical cord analysis. In case of a known familial genetic hearing disorder, it is advisable to analyze newborn siblings for the corresponding genetic defect as soon as possible, to be able to plan and initiate clinical care for the patient as early as possible. It is also extremely important for the parents to obtain clear information about the auditory status of the newborn.

## Introduction

Hearing impairment is the most common sensorineural disorder in humans. Approximately 1–3/1000 newborns are affected by profound hearing impairment to deafness at birth or in the first years of life. The same number of children become deaf before reaching adulthood. The causes of severe hearing impairment are very heterogeneous. Based on the form of the physiological defect, hearing disorders are classified as conductive hearing loss, sensorineural hearing loss, or a combination of both. Reasons for hearing disorders may include environmental factors or viral infections, strong noise sources, ototoxic substances, and genetic causes. Hearing disorders can occur anytime in development and show a progressive course over several years from mild hearing loss to deafness [1, 2].

Approximately ~ 60% of all prelingual hearing disorders are genetic. Inherited hearing disorders are divided into syndromic or non-syndromic; non-syndromic hearing disorders (NSHL) occur in isolation, whereas syndromic hearing disorders (SHL) are associated with additional disorders. Nearly 70% of cases of inherited hearing loss are non-syndromic and predominantly due to sensorineural causes. Of these, about 80% of the cases follow an autosomal recessive (DFNB) and 18% an autosomal dominant (DFNA) mode of inheritance; about 2% are x-linked (DFNX) or mitochondrial linked (MT). A total of 193 gene loci have been described to date, for which 143 genes have been identified so far, and at least 50 genes are still unknown [3].

In most cases, NSHL is due to a monogenetic effect, i.e., one gene defect leads to the disease, but genetic heterogeneity is present. In a few cases, however, defects in multiple genes appear to be required to explain the pattern of inheritance.

Like NSHL, SHL has many different genetic causes. Syndromic hearing disorders are associated, for example, with malformations of the outer ear, inner ear, or petrous bone, and often are associated with other organic diseases, such as thyroid, renal dysfunction, or eye disease. To date, approximately 400 syndromes have been identified that are associated with hearing disorders. In many of these syndromes, hearing loss is mild to moderate and fluctuating, and some of these syndromes are very rare.

The locus DFNB1 [MIM 220290], localized in chromosomal segment 13q11-12, is caused by mutations in the gene GJB2 [MIM 121011] [4], which encodes connexin-26, and by deletions in the gene GJB6 (connexin-30) [MIM604418] [5].

Half of all cases of nonsyndromic recessive hearing impairment are due to alterations in this gene locus. This results in a prevalence rate of these heterozygous gene defects, at the DFNB1 gene locus of approximately 1/60 in the general population [6–8].

Pendred syndrome is one of the most common forms of genetic syndromic hearing disorders with about 1–8/100,000 cases. It is inherited in an autosomal recessive manner, clinically characterized by a sensorineural hearing disorder associated with thyroid dysfunction, which, however, occurs only at the beginning or in the middle of the second decade of life. Furthermore, a dilated aqueductus vestibuli (EVA = enlarged vestibular aqueduct) or Mondini dysplasia is present [1, 9]. Until now, changes in the gene SLC26A4 [MIM 605646] have been held responsible for the Pendred/EVA syndrome.

Our experience has shown that parents want to be informed as soon as possible whether their newborn child has the same genetic defect as their child with the genetical disease [10]. Previous studies could demonstrate that increased parenting stress leads to higher rates of behavior problems in both deaf and hearing children [11–14]. Parents of deaf children struggle with both communication difficulties, increased medical care and educational challenges [14, 15]. There are major stressors as concerns about the future of the child endorsed in parents of deaf children [14]. In this context, genetic analysis of umbilical cord tissue offers a simple and straightforward method to clarify the genetic status of the newborn as early as possible.

## Materials and methods

We performed a retrospective analysis of second and third born siblings of genetically positive tested hearing impaired first born children out of 22 families at a quaternary university hospital and associated cochlear implant center, included between 2002 and 2022. All included first-born patients showed a severe hearing impairment in the first two years of life, so that a cochlear implant treatment was necessary.

We performed a genetic analysis of the gene locus DFNB1 in which the genes GJB2 (connexin-26) and GJB6 (connexin-30) are arranged in tandem orientation. In patients who had enlarged vestibular aqueduct on preliminary CI examination by high-resolution thin-slice CT, sequence analysis of the gene SLC26A4 (Pendrin) was performed as an alternative. Primers, PCR, and sequencing conditions were selected according to procedures described before [16, 17].

Immediately after birth of a sibling, a part of the umbilical cord (10–250 mg) was sent sterile to the molecular genetic laboratory of the University ENT Clinic Freiburg and promptly analyzed for the already known genetic defect of the sibling.

This study took place in the Department of Otorhinolaryngology, Head and Neck Surgery at the Implant Center of the University Hospital Freiburg. The study was approved by the Hospital’s Ethics Committee (local Ethics Committee of the Freiburg University) according to the Declaration of Helsinki (Washington, 2002) (Number of Ethics Committee approval, unlimited: (Number: 161/02, updated: 06/03)). Informed consent was obtained from all subjects and/or their legal guardian(s). The data that support the findings of this study are available from the department of otorhinolaryngology (Medical Center Freiburg) but restrictions apply to the availability of these data, which were used under license for the current study, and so are not publicly available. Data are, however, available from the authors upon reasonable request and with permission of the Medical Center Freiburg and the local Ethics Committee of the Freiburg University.

## Results

Bilateral severe hearing loss was diagnosed in 22 first born children in the first months of life. Inner ear malformations could be excluded in these cases by high-resolution computed tomography (CT) of the temporal bone. Two mutations were identified in 16 of the patients being homozygous carriers of the c.35delG mutation in the GJB2 gene (see Table 1). In five families (family 1, 4, 11, 12 and 15) the umbilical cord of two newborns each was analyzed. The patients in families 16 and 20 had early severe hearing impairment but no alteration in the DFNB gene locus, and the umbilical cord samples of the newborn siblings were accordingly not analyzed.

**Table 1:** Mutation analysis at gene locus in early childhood non-syndromic autosomal recessive inherited hearing impairment (wt = wild type; he = heterozygous)

Family	Child number	Genetic testing	Gene locus	Coding for	Nucleotide	Protein	Year of birth	Year of CI surgery (uni- or bilateral)	Congenital duration of deafness (in months)
1	1	Homozygous	DFNB1	Connexin26	c.35delG	p.Gly12Valfs*2	2002	2004 unilateral, 2007 contra-lateral	23
	2	wt			wt	wt	2004	x	
	3	wt			wt	wt	2009	x	
2	1	Homozygous	DFNB1	Connexin 26	c.35delG	p.Gly12Valfs*2	2002	2004 unilateral, 2007 contra-lateral	22
	2	he/wt			c.35delG/wt	p.Gly12Valfs*2/wt	2010	x	
3	1	he/he	SLC26A4	Pendrin		p.Arg409His/p.Leu236Pro	2003	2005 unilateral, 2007 contra-lateral	18
	2	he/wt				p.Arg409His/wt	2005	x	
4	1	he/he	SLC26A4	Pendrin		p.Ser133Thr/c.1197delT, X430	1993	2008 unilateral	Progressive
	2	wt/wt	SLC26A4	Pendrin			2006	2012 unilateral	Progressive
	3	he/wt				p.Ser133Thr/wt	2006	x	
5	1	Homozygous	DFNB1	Connexin 26	c.35delG/c.176_191del16nt	p.Gly12Valfs*2/Frameshift	2002	2005 unilateral, 2008 contra-lateral	33
	2	Wt			wt	wt	2007	x	
6	1	he/he	SLC26A4	Pendrin		p.Thr132Ile/c.1001 + 1G > A	2001	x	Progressive
	2	he/wt				p.Thr132Ile/wt	2006	x	
7	1	Homozygous	DFNB1	Connexin 26	c.35delG	p.Gly12Valfs*2	2002	2006 unilateral, 2009 contra-lateral	44
	2	he/wt			c.35delG/wt	p.Gly12Valfs*2/wt	2006	x	
8	1	Homozygous	DFNB1	Connexin 26	c.35delG	p.Gly12Valfs*2	2005	2007 unilateral, 2008 contra-lateral	16
	2	wt			wt	wt	2008	x	
9	1	Homozygous	DFNB1	Connexin 26	c.35delG	p.Gly12Valfs*2	2006	2008 unilateral, 2009 contra-lateral	21
	2	he/wt			c.35delG/wt	p.Gly12Valfs*2/wt	2008	x	
10	1	Homozygous	DFNB1	Connexin 26	c.35delG	p.Gly12Valfs*2	2009	2010 unilateral, 2011 contra-lateral	11
	2	he/wt			c.35delG/wt	p.Gly12Valfs*2/wt	2011	x	
11	1	Homozygous	DFNB1	Connexin 26	c.35delG	p.Gly12Valfs*2	2010	2011 unilateral, 2013 contra-lateral	12
	2	Homozygous	DFNB1	Connexin 26	c.35delG	p.Gly12Valfs*2	2011	2012 unilateral, 2013 contra-lateral	11
	3	he/wt			c.35delG/wt	p.Gly12Valfs*2/wt	2016	x	
12	1	Homozygous	DFNB1	Connexin 26	c.35delG	p.Gly12Valfs*2	2010	2011 unilateral, 2012 contra-lateral	11
	2	wt			wt	wt	2013	x	
	3	Homozygous	DFNB1	Connexin 26	c.35delG	p.Gly12Valfs*2	2018	2019 bilateral simultaneously	10
13	1	he/he	DFNB1	Connexin 26	c.35delG/c.31_68del14nt	p.Gly12Valfs*2/Frameshift	2009	2012 unilateral	39
	2	wt			wt	wt	2012	x	
14	1	Homozygous	DFNB1	Connexin 26	c.35delG	p.Gly12Valfs*2	2010	2012 unilateral, 2013 contra-lateral	21
	2	wt			wt	wt	2012	x	
15	1	he/he	DFNB1	Connexin 26	c.35delG/c.71G > A	p.Gly12Valfs*2/Trp24Stop	2012	2012 unilateral, 2013 contra-lateral	11
	2	wt			wt	wt	2016	x	
	3	wt			wt	wt	2021	x	
16	1	Homozygous	DFNB1	Connexin 26	wt	wt	2012	2013 unilateral, 2015 contra-lateral	11
	2	wt			x			x	
17	1	he/he	DFNB1	Connexin 26	c.35delG/c.313-326del14nt	p.Gly12Valfs*2/Frameshift	2011	2013 bilateral simultaneously	26
	2	wt			wt	wt	2014	x	
18	1	Homozygous	DFNB1	Connexin 26	c.35delG	p.Gly12Valfs*2	2014	2015 unilateral, 2016 contra-lateral	11
	2	Wild type			wt	wt	2017	x	
19	1	Homozygous	DFNB1	Connexin 26	c.35delG	p.Gly12Valfs*2	2015	2016 bilateral simultaneously	13
	2	he/wt			c.35delG/wt	p.Gly12Valfs*2/wt	2020	x	
20	1	Homozygous	DFNB1	Connexin 26	wt	wt	2015	2017 unilateral, 2018 contra-lateral	29
	2	wt			x		2017	x	
21	1	he/he	DFNB1	Connexin 26	c.35delG/delGJB6-D13S1830	p.Gly12Valfs*2/delGJB6	2018	2020 bilateral simultaneously	14
	2	he/wt			c.35delG/wt	p.Gly12Valfs*2/wt	2021	x	
22	1	he/he	DFNB1	Connexin 26	c.35delG/c.139G > T	p.Gly12Valfs*2/Glu47/Stop	2017	2018 bilateral simultaneously	9
	2	he/he	DFNB1	Connexin 26	c.35delG/c.139G > T	p.Gly12Valfs*2/Glu47/Stop	2021	Planned 2022	

Three first born patients were diagnosed with Pendred syndrome and an enlarged vestibular aqueduct was confirmed radiologically (family 3, 4 and 6). Hearing loss was progressive in these patients. Two different mutations were identified in each of these three patients. The newborn sibling had no mutation in one case. In three cases a heterozygous mutation could be found.

## Discussion

In the case of a proven genetic cause of hearing impairment, the affected families are interested in finding out soon after delivery to what extent their next born child is also affected by hearing impairment. The considerable emotional stress of the parents must also be taken into account in this context [18].

Practical experience shows newborn hearing screening (NHS) with OAE (otoacoustic emissions) or ABR (auditory brainstem response) is not always reliable. Especially in cases of progressive hearing loss, as is typical for Pendred syndrome, an unremarkable finding in newborn hearing screening can lead to a delay in diagnosis and thus treatment [19, 20].

In contrast, our results from eight cases (Table 1) clearly demonstrated that umbilical cord molecular genetic diagnosis is a simple and rapid method to obtain a definite result as soon as possible when genetic causes of hearing disorders are known in the family.

These analyses can be performed within 2–3 days. Only small amounts of the umbilical cord tissue are needed, 10 mg is approximately equivalent to the head of a pin. Ideally, the collection and shipment of umbilical cord tissue or blood is done in collaboration with the obstetrics department and no additional blood test is required.

On the one hand, mutation analysis of the umbilical cord tissue can rule out the disease and spare both the children and their parents other expensive and time-consuming diagnostic procedures. Alternatively, prenatal diagnosis can be applied to know the hearing status of the second child earlier. However, unlike umbilical cord diagnosis, this method poses a risk to both mother and child. A diagnosis one week after birth is early enough for hearing rehabilitation if necessary. If the condition is disclosed, all necessary steps can be taken to efficiently support speech development in the newborn. Furthermore, it is possible for the parents to be clear at an early stage that the hearing impairment is present or not.

## Conclusion

In case of a known familial genetic hearing disorder, it is reasonable and easy to analyze newborn siblings for the corresponding genetic defect by umbilical cord analysis as soon as possible, to be able to plan and initiate clinical care for the patient as early as possible. It is also extremely important for the parents to obtain clear information about the health of the newborn.

## Data Availability

The data of this study are not openly available due to ethical reasons. The data are available from the corresponding author upon reasonable request.
